# The rs11568820 Variant in the Promoter Region of Vitamin D Receptor Gene Is Associated with Clinical Remission in Rheumatoid Arthritis Patients Receiving Tumor Necrosis Factor Inhibitors

**DOI:** 10.3390/genes15020234

**Published:** 2024-02-12

**Authors:** Andrea Latini, Giada De Benedittis, Paola Conigliaro, Chiara Bonini, Chiara Morgante, Maria Iacovantuono, Arianna D’Antonio, Alberto Bergamini, Giuseppe Novelli, Maria Sole Chimenti, Cinzia Ciccacci, Paola Borgiani

**Affiliations:** 1 Department of Biomedicine and Prevention, Genetics Section, University of Rome “Tor Vergata”, 00133 Rome, Italy; a.latini@med.uniroma2.it (A.L.); dbngdi01@uniroma2.it (G.D.B.); chiaramorgante89@gmail.com (C.M.); novelli@med.uniroma2.it (G.N.); 2 Rheumatology, Allergology and Clinical Immunology, Department of Systems Medicine, University of Rome “Tor Vergata”, 00133 Rome, Italy; paola.conigliaro@uniroma2.it (P.C.); chiarabonini04@gmail.com (C.B.); mariaiacovantuono@gmail.com (M.I.); ari.dan91@gmail.com (A.D.); bergamini@med.uniroma2.it (A.B.); maria.sole.chimenti@uniroma2.it (M.S.C.); 3 School of Medicine, Department of Pharmacology, University of Nevada, Reno, NV 89557, USA; 4 IRCCS NEUROMED, 86077 Pozzilli, Italy; 5 UniCamillus, Saint Camillus International University of Health Sciences, 00131 Rome, Italy; cinzia.ciccacci@unicamillus.org

**Keywords:** rheumatoid arthritis, VDR, treatment response

## Abstract

The vitamin D receptor (VDR), binding to the active form of the vitamin, promotes the transcription of numerous genes involved in the proliferation of immune cells, cytokine production and lymphocyte activation. It is known that vitamin D deficiency can influence the risk of developing rheumatoid arthritis (RA) or modulate its disease activity. The aim of this study was to investigate a possible association between the rs11568820 (C > T) polymorphism in the promoter region of VDR gene and the response to therapy with anti-TNF drugs in patients with RA. A total of 178 consecutive Italian patients with RA treated with anti-TNF, naïve for biological therapy, were recruited. Disease activity data were evaluated using specific indices such as DAS28, CDAI and SDAI, measured at the start of therapy and subsequently at 22, 52, 104 and 240 weeks. A statistically significant association emerged between the rs11568820 variant allele of VDR gene and failure to remission assessed by CDAI and SDAI at 52 weeks, and by DAS28, CDAI and SDAI at 104 weeks of follow-up. Furthermore, the variant allele of this polymorphism was observed more frequently in patients who did not undergo sustained remission calculated by CDAI and SDAI. The variant T allele of rs11568820 in VDR gene is associated with a reduced remission rate with anti-TNFα drugs. These data suggest the role of VDR genetic variability in the response to therapy and in the achievement of remission.

## 1. Introduction

Vitamin D is a pleiotropic hormone synthesized through the absorption of the sun’s rays into the skin. Its key role is the regulation of calcium homeostasis and bone turnover by stimulating osteoclastic and osteoblastic cells [1]. Vitamin D, in its active form (calcitriol), acts as a powerful immunoregulator by activating immune cell proliferation, cytokine production and differentiation or activation of lymphocytes that express the vitamin D receptor (VDR) [2]. In particular, it acts as a suppressor of Th1 and Th17 lymphocytes and its deficiency leads to a reduced capacity to turn off T cells, resulting in the production of inflammatory cytokines [3]. 

Several evidences suggest that low-serum vitamin D levels are closely associated with a higher risk of autoimmune diseases [4,5]. For example, the involvement of vitamin D deficiency in increasing the risk of developing rheumatoid arthritis (RA) and of worsening disease activity is known [6]. Therefore, insufficient vitamin D levels, that may be due to both genetic predisposition or environmental factors, could be involved in the onset and progression of autoimmunity. 

VDR is coded by VDR gene, located on chromosome 12, and variations in its activity can significantly alter the physiological functions in which vitamin D is involved [7]. Germlines genetic variants in the VDR gene have been widely investigated and it is known that they could modulate the expression or function of the VDR [8]. SNPs (single-nucleotide polymorphisms) in the VDR gene have been described to be associated with several autoimmune diseases, such as systemic lupus erythematosus, type 2 diabetes, multiple sclerosis, juvenile idiopathic arthritis and RA [9,10,11,12,13]. 

The high prevalence of vitamin D deficiency in patients with autoimmune diseases often leads to vitamin D supplementation. It has been demonstrated that calcitriol has a synergic effect with monoclonal antibody against TNF-α (tumor necrosis factor-α) in inhibiting the pro-inflammatory pathway of Th17 cells in RA [14]; this suggests that vitamin D combined with TNF-i (TNF-inhibitor) could produce a better response in the treatment of RA patients. For this reason, VDR polymorphisms with functional roles could be associated with clinical outcome and achieving remission in patients undergoing TNF-i treatment. Cusato et al. described an association between VDR rs1544410 SNP and clinical remission at three months of TNF-i therapy in patients with inflammatory bowel disease (IBD) [15]. A study in patients with axial spondyloarthritis showed different improvements in C-reactive protein (CRP) and disease activity scores after 3 and 6 months of TNF-i therapy, depending on the genotype of the VDR polymorphisms rs2228570, rs731236 and rs7975232 [16]. 

However, none of these studies have investigated the possible involvement of rs11568820 SNP in TNF-i treatment response. This variant is located in the promoter region and it seems to be involved in modulating the transcription of the VDR gene. Arai H. et al. has demonstrated that the variant allele of this polymorphism could indeed lead to an increase in mRNA expression [17]. The variant allele of this SNP has been described to be associated with an increased risk of developing gout [18], type 2 diabetes [10] and osteoporosis [19]. This genetic polymorphism has also been investigated in association with dexamethasone treatment response in children with acute lymphoblastic leukemia [20] and with 1α-hydroxyvitamin D3 derivatives treatment response in patients with X-linked hypophosphatemic rickets [21]. Moreover, this variant seems to have a significant association with the response to phototherapy in patients with psoriasis [22].

For these reasons, we supposed that rs11568820 polymorphism, involved in the modulation of VDR transcription, could be associated with clinical outcome and achievement of remission in patients undergoing TNF-i treatment. Since there are no data in the literature on the involvement of this VDR polymorphism in TNF-i treatment response, unlike the other main VDR SNPs, we investigated the possible role of rs11568820 as a predictor of drug efficacy in terms of remission and low disease activity (LDA) in a cohort of Italian subjects with RA during the treatment with first-line TNF-i, in particular with Etanercept (ETN) and Adalimumab (ADA).

## 2. Materials and Methods

### 2.1. Sample Collection

Blood samples and medical records of 178 RA patients referred to the Rheumatology Outpatient Clinic at the Department of Systems Medicine (University of Rome Tor Vergata) were retrospectively analyzed (time frame of the enrolment: 2014–2016). RA patients were classified according to the 2010 American College of Rheumatology (ACR)/European League Against Rheumatism (EULAR) classification criteria [23]. Patients were included in the study if they fulfilled the following inclusion criteria: 18 years of age, inadequate response to at least one conventional synthetic (cs) DMARD, including Methotrexate and naïve for biologic treatment. Patients were excluded from the study if they showed impairment of hepatic/renal function, alcohol abuse, recent infection (with the last infection >3 month ago), ongoing history of malignancy (with interval malignancy-free >5 years) or ongoing pregnancy and if they had missing or incomplete data in the follow-up visits. The demographic and clinical description of patients was reported in a previous paper [24].

Patients received the standard recommended doses of TNF-i: subcutaneous injection of Adalimumab (ADA) at 40 mg every two weeks or Etanercept (ETN) at 50 mg every week. The clinical and laboratory findings were evaluated at baseline and at 22, 52, 104 and 260 weeks from the start of TNF-i therapy. In addition, data on vitamin D supplementation were also collected at baseline and at 22, 52, 104 and 260 weeks from the start of TNF-i therapy. The disease activity and clinical response to therapy were assessed using 28-joint disease activity score (DAS28, LDA < 3.2, remission < 2.6), Clinical Disease Activity Index (CDAI, LDA ≤ 10, remission ≤ 2.8), and Simplified Disease Activity Index (SDAI, LDA ≤ 11, remission ≤ 3.3) [25]. The achievement of remission was considered sustained (SUST) when present at least in two different time-points of follow-up and longer than 6 months [26].

Peripheral blood samples were obtained at the time of the first medical evaluation from all included RA patients in order to perform the genetic analyses. All patients were naïve for biological drugs at the time of blood sampling. Rheumatoid Factor (RF) immunoglobulin A (IgA) and immunoglobulin M (IgM) (normal value < 20 IU/L) were quantified by nephelometry using Immage 800^®^ (Beckman Coulter, Fullerton, CA, USA) according to the manufacturer’s guidelines. Anti-citrullinated peptide antibodies (ACPA) were quantified by second-generation commercial enzyme-linked immunosorbent assay (ELISA) kit (QUANTA Lite^®^ CCP IgG, Bedford, MA, USA) (normal value < 20 IU/L). Samples for genetic analysis were stored at −20 °C until they were analyzed. Written informed consent was obtained from all patients. The local ethics committee of the “Policlinico Tor Vergata” (Rome, Italy) approved the study protocol (Approval No. RS162/21).

### 2.2. DNA Extraction and Genotyping

Genomic DNA was isolated from peripheral blood mononuclear cells using a Qiagen blood DNA mini kit (Qiagen, Valencia, VA, USA). Patients were analyzed for rs11568820 polymorphism in VDR gene by allelic discrimination assay with TaqMan technology (Applied Biosystems, Foster City, CA, USA) using 7500 real-time instrument (Applied Biosystems-Thermofisher, CA, USA). In each run of the allelic discrimination assay, we used samples with known genotypes, confirmed by direct sequencing, as genotype controls.

### 2.3. Statistical Analysis

We evaluated a possible association between the selected SNP and the achievement of remission at 22, 52, 104 and 260 weeks from the beginning of the TNF-i treatment, using as clinical parameters the values of DAS28, CDAI and SDAI remission, and the maintenance of remission at 6 months from the beginning of the TNF-i treatment, using as clinical parameter the values of SUST remission. Differences in genotype frequencies between the two groups of patients, those that have achieved and those that have not achieved the remission, were evaluated by Pearson χ2 test. Odds ratios (ORs) with 95% confidence interval (CI) were calculated. A multivariate logistic regression analysis was used to correct the *p*-value for sex and ACPA/RF positivity. Two-tailed *p*-values less than 0.05 were considered statistically significant. Statistical analyses were performed by the SPSS program ver. 19 (IBM Corp., Armonk, NY, USA).

## 3. Results

We retrospectively analyzed rs11568820 SNP in VDR gene in 178 RA patients naïve to biological therapy, from the start of anti-TNFα therapy (ADA/ETA) (Table 1).

**Figure 1:**
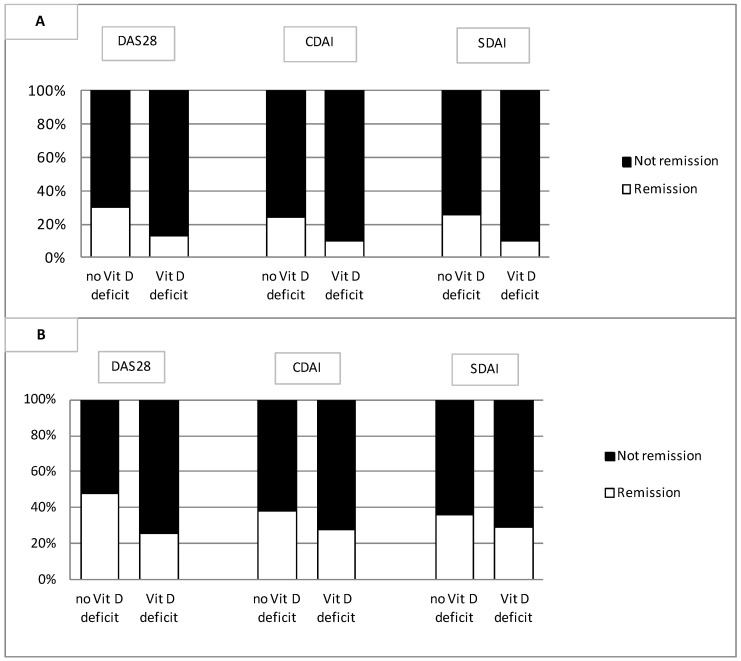
Achievement of remission at T22 (**A**) and T52 (**B**) in RA patients with/without vitamin D deficit. DAS28, Disease Activity Score on 28 joints; CDAI, Clinical Disease Activity Index; SDAI, Simplified Disease Activity Index.

Patients had a long-standing disease (7.40 ± 10.47 years), with 66.9% testing positive for RF and 73.7% testing positive for ACPA. Osteoporosis was present in 27.7% of cases, and vitamin D supplementation was observed in 25.7% of cases because of deficiency. At baseline, patients had a moderate-to-severe disease (DAS28 5.18 ± 1.29). Firstly, we verified a possible relationship between vitamin D levels and drug response. We observed an association between vitamin D deficiency at beginning of the TNF-i treatment and failure to obtain remission assessed in terms of DAS28 (*p* = 0.021), CDAI (*p* = 0.046) and SDAI (*p* = 0.021) at T22 and DAS28 (*p* = 0.016) at T52 (Figure 1).

We investigated the rs11568820 polymorphism of VDR gene in the whole cohort of RA patients. In our population, we observed a frequency of 21.9% for the variant allele, overlapping well with the European (non-Finnish) frequency reported in GnomAD database (19.9%). No associations with clinical characteristics were observed. Moreover, we did not observe associations between this polymorphism and vitamin D deficiency at the beginning of the TNF-i treatment.

Then, we compared the rs11568820 genotypes distribution in relation to LDA (achieved vs. not achieved) and remission (achieved vs. not achieved), evaluated with three different clinimetric indexes of disease activity (DAS28, CDAI, SDAI). All the analyses were performed considering the clinical evaluations during follow up at 22, 52, 104 and 260 weeks after the treatment starting.

We performed the analysis considering the whole cohort of RA patients, independently from the specific TNF-i drug. We did not observe any difference in the distribution of genotypes among patients achieving LDA or not, at any of the follow-up time-points. On the contrary, the rs11568820 SNP appears to associate with the remission in patients treated with TNF-i drugs (Table 2).

In particular, the rs11568820 variant allele was associated with no achievement of CDAI and SDAI remission at 52 weeks (*p* = 0.024 and OR = 0.44, *p* = 0.029 and OR = 0.45, respectively). These associations were confirmed after multiple corrections for sex, age, ACPA/RF positivity and csDMARDs concomitant therapy (Padj = 0.027 and Padj = 0.033, respectively). Moreover, at 104 weeks after the treatment starting, we observed that rs11568820 variant allele was associated with no achievement of remission, considering all the disease activity indexes (*p* = 0.026 and OR = 0.407 for DAS28, *p* = 0.034 and OR = 0.405 for CDAI, *p* = 0.034 and OR = 0.405 for SDAI). All these associations were confirmed after multiple corrections for sex, age, ACPA/RF positivity and csDMARDs (Pcorr = 0.018, Pcorr = 0.030 and Pcorr = 0.030, respectively). We observed a trend of associations with the remission also at 260 weeks after the treatment starting, but this analysis was possible only on 68 patients.

Since the association is confirmed at multiple follow-up times, we evaluated whether the rs11568820 was also associated with the sustained remission at 104 weeks (Table 3).

In accordance with the analyses at different time-points of follow-up, the rs11568820 SNP was also associated with no achievement of sustained CDAI and SDAI remission (*p* = 0.021 and OR = 0.381, *p* = 0.017 and OR = 0.369, respectively). These associations were also confirmed after corrections (Pcorr = 0.027 and Pcorr = 0.027, respectively).

Lastly, we repeated the analysis with each drug separately and we observed that the associations were confirmed mainly in RA patients treated with ETN. Indeed, rs11568820 SNP showed an association with a lack of achievement of remission at 52 and 104 weeks and with sustained remission (Table 4). For patients treated with Adalimumab, we did not observe a statistical significance.

## 4. Discussion

In this study, we evaluated the potential role of rs11568820 VDR SNP on the response to TNF-i treatment in RA patients. We observed that the variant T allele of rs11568820 of the VDR gene appears to be associated with a reduced remission rate during treatment with TNF-i at different time-points of follow-up after the beginning of the treatment. Moreover, we showed that this association is also confirmed with the sustained remission at 104 weeks, suggesting that vitamin D and VDR may have a role not only in achieving the remission, but also in maintaining it over time. 

Up to 40% of patients treated with biological disease-modifying antirheumatic drugs do not adequately respond or lose response over time, making the optimization of choice and durability of these therapies an important priority. Several studies have reported that the occurrence of hypovitaminosis D in RA patients is associated with a reduced response to treatment and a higher disease activity [27,28]. For example, Di Franco et al. showed that, after 12 months of treatment with low doses of corticosteroids and methotrexate, the percentage of remission was significantly lower in RA patients with low-basal vitamin D levels compared to patients with normal-basal vitamin D levels [29]. A significant association between vitamin D levels and remission rates has also been repeatedly reported for anti-TNF treatment, for example, in patients with IBD [30,31]. It is now known that polymorphisms of the VDR gene can lead to functional changes modulating the regulatory effects of vitamin D, as well as in the immune response. Indeed, associations between clinical response or remission during anti-TNFα therapy and VDR polymorphisms has been reported [15,16], but to our knowledge, this is the first study that has investigated the possible involvement of rs11568820 SNP in TNF-i treatment response. 

The rs11568820 SNP is located in a regulatory region upstream of the VDR promoter transcription start site and it is potentially able to influence its expression through differential binding to the transcription factor CDX-2 (caudal-type homeobox protein 2). A functional study on colon-derived Caco-2 cell line showed that CDX-2 binds the variant allele with the strongest affinity, effecting a 15% increase in transcriptional activity of the VDR gene [17]. However, CDX2 has tissue-specific expression, mainly in intestinal tissue, and is absent in white blood cells [32]. Indeed, studies conducted on monocytes [32] and peripheral blood mononuclear cells [19] have observed an opposite trend, with significantly lower VDR levels in the variant genotype compared to the other genotypes. In the absence of CDX2 transcription factor, the variant allele therefore appears to negatively affect VDR expression levels. The decrease in VDR expression could explain the association between the rs11568820 variant allele and a lack of remission in RA patients treated with TNF-i. Indeed, data in the literature reported that vitamin D, combined with TNF-I, seems to produce a better response in the treatment of RA patients. Based on our results, our hypothesis is that the rs11568820 may negatively influence the VDR expression and this could in part explain the lack of remission. In support of the role of vitamin D in achieving remission, we have also observed an association between vitamin D deficiency at the beginning of the TNF-i treatment and a failure to gain remission. Indeed, data from the literature demonstrate that vitamin D is involved in the mechanisms of the regulation of the immune system, the synthesis of cytokines, and modulate cellular apoptosis [33]. Vitamin D levels display an inverse correlation with RA disease activity measured by DAS28 and it is associated with a worse quality of life, wherein disability progression in over one year was predicted in a study with 645 early-RA patients [34,35,36]. Consistent with these data, VDR genetic variability and the individual’s vitamin D status could therefore represent two important factors to take into account for the achievement and maintenance of remission in RA patients treated with TNF-i. Future studies on large cohorts could enable the stratification of patients based on the presence of biomarkers useful in therapy selection. The presence of the rs11568820 polymorphic variant in the promoter region of vitamin D and vitamin D deficiency could be considered to eventually guide treatment towards drugs with a different mechanism of action compared to anti-TNF agents. 

A limitation in our study is the absence of data about VDR expression levels and vitamin D values in RA patients at different follow-up sessions, because the analysis is retrospective and the information was not collected at the time of recruitment. 

In conclusion, our results showed for the first time the association of rs11568820 polymorphism in the VDR gene and remission rate in RA patients treated with TNF-i drugs. These data support the role of VDR genetic variability in vitamin D function and suggest a contribution of this variability in the response to therapy and in the achievement of remission.

## Figures and Tables

**Table 1:** Demographic and clinical data of 178 patients with rheumatoid arthritis included in the study.

Age (years ± SD)	53.55 ± 12.87
Disease duration (years ± SD)	7.40 ± 10.47
Sex (female)	76.4%
Etanercept/Adalimumab (%)	57.3%/42.7%
RF positivity (%)	66.9%
ACPA positivity (%)	73.7%
Radiographic Erosions (%)	61.4%
Osteoporosis (%)	27.7%
Vitamin D Supplementation (%)	25.7%
DMARDs (%)	80.0%
PDN (%)	56.0%
Baseline DAS28	5.18 ± 1.29
Baseline CDAI	26.24 ± 13.64
Baseline SDAI	28.42 ± 21.01

Data presented as number of patients (%) or mean ± Standard Deviation. RF, Rheumatoid Factor; ACPA, anti-citrullinated peptide antibodies; csDMARDs, conventional synthetic disease-modifying antirheumatic drugs; PDN, prednisone; DAS28, Disease Activity Score on 28 joints; CDAI, Clinical Disease Activity Index; SDAI, Simplified Disease Activity Index.

**Table 2:** Association analysis between rs11568820 VDR polymorphism and remission achievement in RA patients treated with TNF-inhibitors.

T22							
	Remission	Wt	Hz	Hom Var	*p* *	OR (95% CI)	P*corr*^+^
DAS28 remission	No	76	44	5	0.774	1.101 (0.572–2.117)	0.349
	Yes	31	20	2			
CDAI remission	No	81	51	5	0.623	0.834 (0.406–1.716)	0.342
	Yes	26	13	2			
SDAI remission	No	78	51	5	0.365	0.720 (0.354–1.468)	0.255
	Yes	29	13	2			
**T52**							
	**Remission**	**Wt**	**Hz**	**Hom var**	*p* *	**OR (95% CI)**	**P*corr*^+^**
DAS28 remission	No	54	29	4	0.865	0.945 (0.494–1.808)	0.097
	Yes	45	24	2			
CDAI remission	No	58	40	5	**0.024**	0.440 (0.214–0.905)	**0.027**
	Yes	41	13	1			
SDAI remission	No	58	40	5	**0.029**	0.451 (0.219–0.929)	**0.033**
	Yes	40	13	1			
**T104**							
	**Remission**	**Wt**	**Hz**	**Hom var**	*p* *	**OR (95% CI)**	**P*corr*^+^**
DAS28 remission	No	48	34	2	**0.026**	0.407 (0.183–0.908)	**0.018**
	Yes	36	9	2			
CDAI remission	No	53	35	3	**0.034**	0.405 (0.173–0.948)	**0.03**
	Yes	31	8	1			
SDAI remission	No	53	35	3	**0.034**	0.405 (0.173–0.948)	**0.03**
	Yes	31	8	1			

wt: wild-type, hz: heterozygous, Hom var: homozygous variant, OR: odds ratio, CI: confidence interval. * *p*-value was calculated Wt vs. Hz + Hom var. Pcorr+: correction for sex, age, ACPA/RF positivity and DMARDs. Significant *p*-values are reported in bold.

**Table 3:** Association analysis between rs11568820 VDR polymorphism and sustainable remission in RA patients treated with TNF-inhibitors.

	Sust Remission	Wt	Hz	Hom var	*p* *	OR (95% CI)	P*corr*^+^
DAS28 remission	No	56	38	5	0.080	0.521 (0.249–1.088)	0.092
	Yes	35	13	1			
CDAI remission	No	61	40	5	**0.021**	0.381 (0.166–0.878)	**0.027**
	Yes	32	8	1			
SDAI remission	No	59	40	5	**0.017**	0.369 (0.160–0.850)	**0.027**
	Yes	32	8	1			

wt: wild-type, hz: heterozygous, Hom var: homozygous variant, OR: odds ratio, CI: confidence interval. * *p*-value was calculated Wt vs. Hz + Hom var. Pcorr+: correction for sex, age, ACPA/RF positivity and DMARDs. Significant *p*-values are reported in bold.

**Table 4:** Association analysis between rs11568820 VDR polymorphism and remission achievement in RA patients treated with Etanercept.

T52							
	Remission	Wt	Hz	Hom var	*p* *	OR (95% CI)	P*corr*^+^
DAS28 remission	No	33	18	4	0.397	0.703 (0.311–1.591)	0.705
	Yes	32	13	2			
CDAI remission	No	35	26	5	**0.002**	0.226 (0.083–0.614)	**0.017**
	Yes	30	5	1			
SDAI remission	No	35	26	5	**0.003**	0.234 (0.086–0.637)	**0.019**
	Yes	29	5	1			
**T104**							
	**Remission**	**Wt**	**Hz**	**Hom var**	*p* *	**OR (95% CI)**	**P*corr*^+^**
DAS28 remission	No	28	20	2	**0.045**	0.377 (0.143–0.991)	0.231
	Yes	27	6	2			
CDAI remission	No	33	22	3	**0.027**	0.300 (0.100–0.902)	0.092
	Yes	22	4	1			
SDAI remission	No	33	22	3	**0.027**	0.300 (0.100–0.902)	0.092
	Yes	22	4	1			
**Sustainable remission**							
	**Remission**	**Wt**	**Hz**	**Hom var**	*p* *	**OR (95% CI)**	**P*corr*^+^**
DAS28 remission	No	31	23	5	**0.014**	0.316 (0.124–0.808)	0.229
	Yes	28	7	1			
CDAI remission	No	36	25	5	**0.009**	0.250 (0.085–0.735)	**0.036**
	Yes	24	4	1			
SDAI remission	No	35	25	5	**0.007**	0.243 (0.083–0.716)	**0.036**
	Yes	24	4	1			

wt: wild-type, hz: heterozygous, Hom var: homozygous variant, OR: odds ratio, CI: confidence interval. * *p*-value was calculated Wt vs. Hz + Hom var. Pcorr+: correction for sex, age, ACPA/RF positivity and DMARDs. Significant *p*-values are reported in bold.

## Data Availability

Data are available on request.
